# Importance of Close Follow-Up in the Fetus with Premature Atrial Contractions Accompanied by Atrial Septal Aneurysm: A Case Report

**DOI:** 10.1155/2013/391085

**Published:** 2013-12-22

**Authors:** Yilmaz Yozgat, Ayhan Kilic, Cem Karadeniz, Rahmi Ozdemir, Onder Doksoz, Timur Mese, Nurettin Unal

**Affiliations:** ^1^Department of Pediatric Cardiology, Izmir Dr. Behcet Uz Children's Hospital, 35210 Izmir, Turkey; ^2^Department of Pediatric Cardiology, Gulhane Medical Academy, Ankara, Turkey

## Abstract

Rhythms that derive from parts of atria other than the sinus node are called premature atrial contractions (PACs). Vast majority of fetal PACs are idiopathic. Fetal PACs usually have a good prognosis and disappear spontaneously during pregnancy or after delivery. Development of fetal tachycardia or fetal bradycardia is rarely reported during follow-up of fetuses diagnosed with PACs. To the best of our knowledge, coexistence of tachycardia and bradycardia leading to hemodynamic impairment has not yet been reported. We present a fetus diagnosed with PACs and atrial septal aneurysm (ASA) on the 23rd week of gestation proceeding to fetal bradycardia and fetal tachycardia and consequently hemodynamic impairment. We suggest closer follow-up of fetuses with PACs accompanied by ASA.

## 1. Introduction

Rhythms originating from atrial regions other than the sinus node are defined as premature atrial contractions (PACs). Vast majority of fetal PACs are idiopathic. Fetal PACs usually have a good prognosis and disappear spontaneously during pregnancy or after delivery [1]. PACs may rarely develop secondarily to underlying structural abnormalities. Presence of an atrial septal aneurysm (ASA) is the most commonly reported structural anomaly in fetuses [2, 3].

Development of fetal tachycardia or fetal bradycardia is rarely reported during follow-up of fetuses diagnosed with PACs [2–4]. To the best of our knowledge, coexistence of fetal tachycardia and bradycardia leading to hemodynamic impairment in the same fetus possibly due to coexisting PAC and ASA has not yet been reported. We present a fetus diagnosed with PACs and ASA on the 23rd week of gestation proceeding to fetal bradycardia and fetal tachycardia and consequently hemodynamic impairment.

## 2. Case Report

A 29-year-old woman was referred for evaluation of suspected fetal arrhythmia in the 23rd week of her pregnancy. Obstetric ultrasonography was negative for fetal anomalies. Fetal echocardiography showed an enlarged and mobile ASA extending as much as 50% into the left atrium and frequent PACs. She was scheduled for weekly hospital visits to allow for early detection of potentially serious fetal arrhythmias.

**Figure 1:**
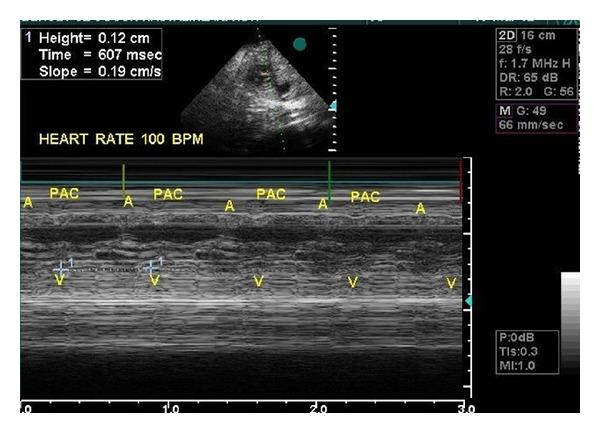
M-mode fetal echocardiogram showing bigeminal PACs associated with a fetal ventricular rate of 80–100 bpm (the A-PAC interval is markedly shortened).

**Figure 2:**
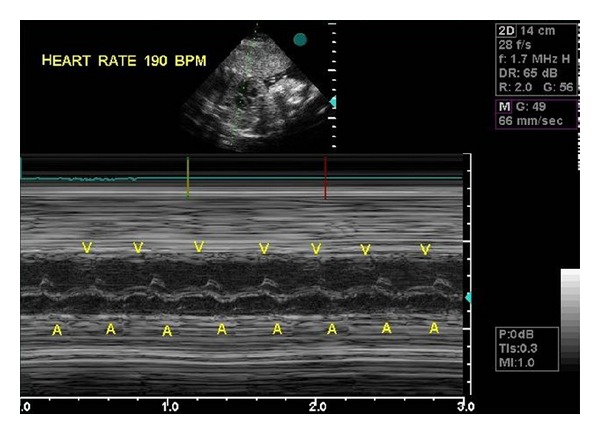
M-mode fetal echocardiogram shows sinus tachycardia of 180–190 per minute.

Beginning from 30th week of gestation, brief but frequent (lasting for 3–5 min with 5 min intervals) episodes of fetal bradycardia due to blocked bigeminal PACs were observed, with ventricular rates varying between 80 and 100 bpm. Differential diagnosis between paradoxical bradycardia due to blocked PACs and 2 : 1 atrioventricular (AV) block seen in fetuses of women with collagen tissue disease was done using fetal echocardiography. While the interval between a normal sinus beat (A) and the following nonconducted atrial contraction is normal or only mildly irregular in 2 : 1 AV block, the A-PAC interval is markedly shortened in atrial bigeminy blocked in the refractory AV node [5]. The A-PAC interval was narrow in our case (Figure 1) and the mother did not have any history or findings compatible with connective tissue disease. Anti-Ro (SS-A) and anti-La (SS-B) antibodies were negative. Fetal bradycardia episodes continued until the 35th week of gestation without any signs of hemodynamic derangement to be followed by a continuous sinus tachycardia of 180–190 bpm (Figure 2). Caesarean section delivery was done on the 37th week of gestation to avoid perinatal morbidity due to increasing tricuspid valve insufficiency and left ventricular dysfunction (shortening fraction 25%). Heart rate of the healthy newborn was 140 bpm and shortening fraction was 30% after delivery. Rare PACs were seen on ECG. Two months after delivery, the PACs disappeared and the ASA was seen by echocardiography to adhere to the septum secundum. The infant is free of arrhythmias after one year of follow-up.

## 3. Discussion

Early in the development of the interatrial septum, a thin membranous septum primum appears in the fetal atrium and separates the atria. Later in pregnancy, the thicker septum secundum develops and grows alongside the septum primum. If the septum primum tissue grows extensively or its supporting tissue is scarce, the loose and wide septum primum flap becomes mobile [6]. If the enlarged septum primum flap (foraminal flap) extends as much as 50% into the left atrium in intrauterine life, this tissue is defined as atrial septal aneurysm (redundant septum primum flap, foramen ovale aneurysm) [7].

PACs are diagnosed by the relationship between concurrent atrial and ventricular contractions in M-mode and/or pulsed-wave Doppler echocardiography [3]. PACs may be conducted or not conducted to the ventricles through the AV node and His bundle. PACs not conducted to the ventricles are named blocked PACs. Nonconducted ectopic beats alternating with normal sinus beats constitute the so-called blocked atrial ectopic rhythm. As only transmitted atrial currents lead to ventricular contraction, heart rate (ventricular rate) may decrease to half the normal heart rate [5]. Fetal heart may provide metabolic needs of the fetus unless the heart rate decreases below 60 bpm [8]. In our case, heart failure did not develop during 5 weeks of fetal bradycardia.

Papa et al. reported ASA in 93 (7.6%) of 1223 fetuses, with 36% of them being associated with PACs [2]. They concluded that the pressure exerted by the redundant septum primum on the atria caused the ectopic rhythm, which closely resembles the situation in our case.

Immediately after birth, normalized pulmonary circulation causes the pulmonary venous return and the left atrial pressure to increase and thereby the pressure difference between the right and left atria becomes prominent. As a result of this increased pressure difference between the atria, the foraminal flap of the septum primum adheres to the septum secundum and its movement is limited, eliminating the cause for the PACs [4]. In our case too, PACs disappeared simultaneously with the disappearance of ASA two months after birth.

Martucci et al. reported a fetus with pseudobradycardia due to trigeminal PACs on the 33rd week of gestation. Their case also showed a normal rhythm immediately after delivery; however, contrary to our case, trigeminal PACs and supraventricular tachycardia (SVT) developed 11 days after birth [9].

Fetal tachycardia is defined as fetal heart rates being more than 180 bpm [2]. Fetal tachycardia may vary between benign sinus tachycardia and SVT which may lead to hydrops fetalis. Fetal sinus tachycardia is defined as a fetal heart rate not exceeding 210–220 bpm with a well-preserved AV conduction. It is a benign condition not requiring treatment expected to improve spontaneously during the first year of life [10]. In our case, however, fetal bradycardia followed by incessant fetal sinus tachycardia caused gradually increasing tricuspid valve insufficiency and ventricular dysfunction and necessitated early delivery.

We conclude that fetuses with PAC and ASA should be followed up closely until the resolution of the rhythm and disappearance of the ASA either pre- or postnatally.
